# Circulating apo 2L levels decreased in hepatitis C with the pegilated interferon-2 alpha treatment

**DOI:** 10.1186/1939-4551-8-S1-A8

**Published:** 2015-04-08

**Authors:** Ata Nevzat Yalcin, Arzu Didem Yalcin, Betul Celik, Sukran Kose, Ayhan Cekin, Derya Seyman, Saadet Gumuslu

**Affiliations:** 1 Department of Infectious Diseases and Clinical Microbiology, Akdeniz University, 07070, Antalya, Turkey; 2 Internal Medicine, Allergy and Clinical Immunology, Genomics Research Center, Academia Sinica,11529, Taipei, Taiwan; 3 Department of Laboratory Medicine and Pathology, Mayo Clinic in Jacksonville, USA; 4 Department of Infectious Diseases and Clinical Microbiology, Allergy and Clinical Immunology Unit, Tepecik Education and Research Hospital. Izmir, Turkey; 5 Department of Gastroenterology, Antalya Training Hospital, Antalya, Turkey; 6 Department of Infectious Diseases and Clinical Microbiology, Antalya Education and Research Hospital, Turkey; 7 Department of Medical Biochemistry, Faculty of Medicine, Akdeniz University, 07070, Antalya, Turkey

## Background

Chronic hepatitis C (HCV) infects approximately 170 million people and causes more than 350 000 deaths every year. Information regarding pathogenetic mechanism of acute hepatitis C infection is limited. Following innate immune activation, cellular immunity, including natural killer (NK) cell activation and antigen-specific CD8 cell proliferation occurs. CD8+ T lymphocytes directly kill infected cells via direct cell-cell contact, and release antiviral cytokines (e.g. IFN, TNF)

## Methods

Eleven HCV-treatment naive HCV infected patients were treated with weight-based ribavirin daily in addition to either weekly pegIFN alfa-2b at 1.5 ug/kg, weekly pegIFN alfa-2a, or albinterferon alfa-2b at 900mcg every 2 weeks. All patients gave written informed consent approved by the Institutional Review Board prior to enrollment in the studies. Intensive serum monitoring was completed at study visits day 0 (pretreatment), weeks 4, 6 and 12.

## Results

In this present study, we aimed to investigate the relationship between IFN treatment response, HCV viral load and sApo 2L levels. Eleven HCV-treatment naive HCV infected patients were treated with pegIFN alfa-2a. Intensive serum circulating Apo 2L levels were monitered at study visits day 0 (pretreatment), weeks 4, 6 and 12.HCV-RNA and sApo 2L levels decreased gradually with PegIF-α 2 treatment and the differences were significant between day 0 and 4^th^ week (p=0.001, p<0.005 and p=0.01, p<0.005 respectively); between day 0 and 12th week (p=0.001, p<0.005 and p=0.001, p<0.000 respectively); between 6th week and 12th week (p=0.01, p<0.05 and p=0.01, p<0.05 respectively).

## Conclusions

We suggest that, decreased level of circulating Apo 2L may reflect its increased binding to its ligand expressed on hepatocyte or lymphocyte under the influence of PegIFN treatment.

